# Paramedic information needs in end-of-life care: a qualitative interview study exploring access to a shared electronic record as a potential solution

**DOI:** 10.1186/s12904-019-0498-2

**Published:** 2019-12-05

**Authors:** Rebecca Patterson, Holly Standing, Mark Lee, Sonia Dalkin, Monique Lhussier, Catherine Exley, Katie Brittain

**Affiliations:** 1 Research and Innovation Services, Northumbria University, Pandon Building, Camden Street, Newcastle upon Tyne, NE2 1XE UK; 2 Department of Nursing, Midwifery and Health, Northumbria University, Newcastle upon Tyne, UK; 3 St Benedict’s Hospice and Specialist Palliative Care Centre, Sunderland, UK; 4 Department of Social Work, Education and Community Wellbeing, Northumbria University, Newcastle upon Tyne, UK; 5 Faculty of Medical Sciences, Newcastle University, Newcastle upon Tyne, UK

**Keywords:** Allied health personnel, Paramedic, End-of-life, Information technology, Decision making

## Abstract

**Background:**

Limited access to, understanding of, and trust in paper-based patient information is a key factor influencing paramedic decisions to transfer patients nearing end-of-life to hospital. Practical solutions to this problem are rarely examined in research. This paper explores the extent to which access to, and quality of, patient information affects the care paramedics provide to patients nearing end-of-life, and their views on a shared electronic record as a means of accessing up-to-date patient information.

**Method:**

Semi-structured interviews with paramedics (*n* = 10) based in the north of England, drawn from a group of health and social care professionals (*n* = 61) participating in a study exploring data recording and sharing practices in end-of-life care. Data were analysed using thematic analysis.

**Results:**

Two key themes were identified regarding paramedic views of patient information: 1) access to information on patients nearing end-of-life, and 2) views on the proposed EPaCCS. Paramedics reported they are typically unable to access up-to-date patient information, particularly advance care planning documents, and consequently often feel they have little option but to actively treat and transport patients to hospital – a decision not always appropriate for, or desired by, the patient. While paramedics acknowledged a shared electronic record (such as EPaCCs) could support them to provide community-based care where desired and appropriate, numerous practical and technical issues must be overcome to ensure the successful implementation of such a record.

**Conclusions:**

Access to up-to-date patient information is a barrier to paramedics delivering appropriate end-of-life care. Current approaches to information recording are often inconsistent, inaccurate, and inaccessible to paramedics. Whilst a shared electronic record may provide paramedics with greater and timelier access to patient information, meaning they are better able to facilitate community-based care, this is only one of a series of improvements required to enable this to become routine practice.

## Key statements

What is already known about the topic?

- Though paramedics are increasingly dealing with end-of-life, they often feel uncertain about how to best care for dying individuals.
- Limited patient information is a key factor leading paramedics to adopt a ‘default’ approach of transferring patients to hospital.
- Recently there have been increased efforts to educate and up-skill paramedics in palliative and end-of-life care, but comparatively there has been little focus on improving the availability of patient information provided to them prior to/during their visit.

What this paper adds

- Paramedics are heavily reliant on, but often struggle to understand, advance care planning documents to inform decision-making when providing care at end-of-life.
- Poor access to advance care planning can leave paramedics feeling ill-equipped and concerned about facilitating community-based care.
- Access to a shared electronic patient record may offer a solution, however, paramedics acknowledge barriers exist which may prevent the implementation of, and/or engagement with, such a record.

Implications for practice

- Education for all healthcare professionals on writing high quality advance care planning documents is vital.
- Development of an effective and consistent system will improve the quality of decision-making; and facilitate the fulfilment of patients’ end-of-life wishes.
- Information must strike a balance between being comprehensive, and yet succinct enough, to facilitate decision-making in an emergency situation.

## Background

The role of the ambulance service has changed considerably over the last decade, with paramedics increasingly involved in community-based care [1, 2]. As part of this, and in spite of a reported lack of confidence [1–3], paramedics have become increasingly involved in delivering end-of-life care, supporting patients who are ‘considered to be in the last stage of their lives’ including individuals living with an advanced, progressive, incurable illness’ [4]. Limited access to patient information is a key concern for paramedics taking on these responsibilities [1, 5]. Paramedics are unable to access patient records and are restricted in their ability to consult with the patient’s usual care team out-of-hours [5–7]. As a result, they are often required to make difficult end-of-life care decisions based on limited information or input from other healthcare professionals [2, 5, 7]. The information available to paramedics can be brief and potentially misleading [7], meaning that decisions have to be based on the limited details communicated by the dispatch team or family members/carers present on arrival, patient-held paper-based care documents, and their immediate medical assessment [5, 7, 8]. Some of the most important patient-held care documents are advisory forms produced through Advance Care Planning (ACP), a process of formal decision-making ideally with healthcare professionals that aims to document patient wishes, values, and preferences for future care (including the Do Not Attempt Cardiopulmonary Resuscitation order - a single-page advisory form signed by a doctor stating that a patient does not want to be resuscitated often issued to patients close to death or with an irreversible condition who are unlikely to benefit from this procedure, and the Emergency Health Care Plan - an advisory document providing an overview of the patient’s diagnosis, medications, care limitations, and desirable actions in potential emergencies) [9–12]. Though ACP is recommended as best practice it remains a voluntary process, thus care plans are not completed by every person being cared for at end-of-life [10, 13]. In cases where ACP has been completed, paramedics routinely struggle to understand and/or trust the validity of this paper-based documentation, leaving them uncertain about how best to proceed [1, 3, 6]. Research from the UK and beyond reveals that limited time and care options, and fear of wrongdoing and subsequent litigation, leaves paramedics feeling obliged to revert to the ‘default’ approach of transporting the patient to hospital [1, 7, 14, 15]. While this approach affords paramedics a level of protection, it has been widely criticised as a costly and inappropriate response, which can defy patient wishes and place vulnerable individuals at risk of physical and psychological harm [3, 16–18]. UK-based alternatives to hospital care, such as referral to a general practitioner (GP), a community palliative care team, or transfer to a hospice, can be challenging to facilitate out-of-hours [3, 5, 7]. Ambulance services have recently invested in improving end-of-life care training, to initiate a shift away from the historical response of transporting patients to hospital toward prioritisation of community-based care [2, 3, 6].

Mirroring the movement toward electronic patient records in the US and Europe [19, 20], the NHS is tasked with innovating the way it uses health data and technology to improve health outcomes and service quality. In 2014, the National Information Board published a Framework for Action detailing how the NHS intends to achieve digital, real-time, and interoperable health records by 2020 [21]. The planned changes will enable patients to have more control and ensure that health and social care professionals are able to access all data, information, and knowledge needed to facilitate good patient care. To meet this challenge, key barriers to the successful sharing of health data between different health and social care professionals need to be explored. The ambulance service face many barriers to efficient information sharing, as paramedics work across localities and interact with many Information Technology (IT) systems [5]. The South East Coast Ambulance Service have sought to overcome this by establishing information-sharing relationships with other healthcare providers, permitting their paramedics access to vital patient information [2]. One of the most widely used technological innovations of this type in the UK is an Electronic Palliative Care Coordination system (EPaCCS) [22, 23]. These are electronic registers that allow various healthcare professionals, including paramedics, to access and share information pertinent to the end-of-life in real time, such as diagnosis, prognosis, preferences, and advance care plans [6, 22, 23]. Early evidence indicates that EPaCCS have a significant impact on practice, reducing unnecessary hospital admissions and interventions, thus enabling more patients to die in their preferred place [22–25].

This paper investigates the extent to which access to, and quality of, patient information affects the care paramedics provide to patients nearing end-of-life, and their views on access to a shared electronic record as a means of improving the information flow around end-of-life care.

## Methods

This paper reports on paramedic data from a study exploring health and social care professionals' (*n* = 61) experiences of data recording and sharing practices in end-of-life care. Paramedic participants (*n* = 10) were recruited through a call for volunteers circulated by email within an ambulance service in the north of England serving a geographically diverse region with a population of 2.6 million. Given the practical issues of recruiting shift-working emergency service personnel to research, an opportunistic sampling strategy [20] was used, meaning all who expressed an interest in the study were interviewed. The sample encompasses individuals employed in different roles within the paramedic profession, including research paramedics, rapid response paramedics, and advanced care practitioners. To preserve anonymity, we refer to all participants using the term ‘paramedic’ throughout this paper.

### Interviews

Participants were interviewed face-to-face (*n* = 5) or by telephone (n = 5), depending on personal preference and geographical location. Most participants were interviewed one-to-one (*n* = 8), however, two individuals participated together in a dyadic interview. A semi-structured interview approach was adopted, informed by a topic guide developed collaboratively by KB, HS, and RP. This document was revised after each interview and amended to incorporate new avenues of enquiry, where necessary. Questions focused on individual’s experience of providing care to persons  nearing end-of-life, particularly their ability to access and share data on this patient group, and the consequent impact on their decision-making processes. Participants were also questioned on their views of the proposed EPaCCS, as a way of accessing, recording, and sharing patient information.

All interviews were conducted by RP and KB in June and July 2018. Participants who expressed interest in taking part were given a research information sheet. Written consent was provided by all participants prior to the interview. Interviews lasted an average of 52 min and were digitally recorded, transcribed verbatim, and anonymised, with pseudonyms used where appropriate to protect participant identity. Ethical approval for this study was obtained from the Health Research Authority (REC reference: 17/LO/2100).

### Analysis

Interview transcripts were analysed using thematic analysis, following the five steps outlined by Braun and Clarke (2006): 1) data familiarisation, 2) generation of preliminary codes, 3) search for themes, 4) review themes, 5) define and name themes [26]. The data management software NVivo 12 was used to develop and refine a coding scheme. Analysis was an iterative process, researchers engaged in continual process of reading and rereading the transcripts, with early transcripts being revisited in light of analysis from later interviews. Several themes and subthemes were generated to reflect the nuances in the data [27]. The themes and subthemes identified through the coding process were examined, summarised, and refined, in a process informed by relevant literature. Coding was primarily completed by RP, a proportion of the transcripts (20%) were also coded independently by HS and KB, before coming together with RP to compare analysis and agree themes. Analytic insight was also sought from the wider research team (including SD, MLh, and ML). This paper predominantly focuses on a key theme within the data set - ‘patient information’; presenting data on paramedic ability to access, record, and share patient information, and thoughts on how this may be affected, positively or negatively, if an EPaCCS was introduced. The headings included in this paper have been selected to guide the reader through the main themes that arose around this topic within the interviews.

## Results

The final sample comprised 9 men and 1 woman, all white-British, with an average of 13 years of experience working in the ambulance service in various roles, including research paramedic, rapid response paramedic, and advanced care practitioner. Two key themes emerged from paramedic interviews: Access to information on patients nearing end-of-life and views on the proposed EPaCCS. Each are outlined in more detail below with illustrative quotations.

### Access to information on patients nearing end-of-life

Paramedics highlighted access to timely and appropriate patient information as being crucial for safe and effective care. However, access to information (paper-based or electronic) is a challenge for health care professionals working in the community. Paramedics expressed that information currently available to them when responding to a call is limited, typically comprising name, age, and condition. In situations where the patient is known to be receiving end-of-life care, paramedics reported sometimes being forewarned, however, this is rare and occurs only when this information has been disclosed by the caller or previously logged by the call centre. Interviewees described the information they received as varied in detail and accuracy, leaving them feeling as though they were often entering challenging situations ‘blind’:

… you’re going into that house, that situation, that nursing home with very limited information unless there’s something flagged with the control centre [ … ] now, there are more things being logged with the control centres. So advance care plans and things like that … Unfortunately, as a paramedic, you learn to take what you’re given prior to getting there, with a pinch of salt, because so many jobs you go to, you don’t get the full picture before you get there. (Paramedic1)

With the exception of those trained to the level of Advanced Practitioner, paramedics stated that they are not permitted access to patient records. Consequently, they reported having to seek verbal information, where possible, from the patient or other individuals present, or attempt to access patient-held documentation. Time permitting, they may also attempt to contact the patient’s community-based care team to glean up-to-date information such as medical history. Acquiring information in this ad-hoc fashion means there is potential for key information to be overlooked or miscommunicated:

If there’s no nursing notes on scene and we can’t rely on the family, then we’ve actually not got anything unless we can get hold of the GP. On weekends or bank holidays it’s usual that we can’t get hold of any sort of information other than what’s lying around the house. (Paramedic5).

Upon establishing that the patient was receiving end-of-life care, interviewees stated it is common practice to seek ACP documentation. While in theory these forms were seen as vital to decision-making, as they provide detail on patient wishes regarding the limits of future care, the extent to which they were felt to be useful in reality was limited by several issues, including: time taken to access, level of detail, and accuracy. Locating accurate, signed, and in-date DNACPR forms was acknowledged to be problematic:

We look for the signature and the date that [the DNACPR form] was written. I think you’ve got a year from the signature date … That’s what the crews tend to look at. They are usually handled by the family. Sometimes they can’t find them. They say, “We think we’ve got one. It’s in a drawer somewhere.” … The family are insisting there’s a DNACPR but they can’t produce it. The crew are like, “What do we do here? This is a problem. It’s not readily available to read or look at.” … We need to see [the DNACPR] … it kind of leaves you in a really awkward position. “Should I or should I not [resuscitate]?” You know? (Paramedic10).

Similarly, access to EHCPs was deemed a rarity, with one interviewee noting it is ‘very hit and miss whether a patient has one or not’ (Paramedic7). Paramedics acknowledged that even when available, EHCPs are typically too abstract and vague to aid decision-making in emergency situations:

… to me, 9 times out of 10 [EHCPs] are not worth the paper that they’re written on [… they’ve been produced by] someone, a GP, say, and the family members [who] know the patient’s history, the patient might have been there, and they’ve come up with a plan between them … knowing all the facts, what they are writing makes perfect sense. When I come in blind, and I’m reading that end-of-life care plan, I’m trying to figure out what they actually mean because it’s not always written in black and white. A paramedic coming in needs to read this in 30 seconds and he needs to make a decision, and it’s not really interpretable … What they need to do is sit around the table and say, “Right, I’m going to write this care plan for paramedics.” (Paramedic4).

Limited access to accurate and explicit patient information was acknowledged to have significant consequences for both paramedics and the individuals in their care. Paramedics noted that poor access to good quality patient information placed them in a challenging position regarding how best to care, and make decisions, for patients nearing end-of-life. Without such documentation, paramedics lacked the confidence to make decisions that deviated from the standard treatment protocol of resuscitating and transferring to hospital. Interviewees acknowledged that fear of litigation meant paramedics often ‘err on the side of caution’ (Paramedic2) when they have little and/or poor patient information, transferring the patient to hospital even if this course of action goes against the patient’s wishes or is deemed potentially inappropriate:

… your decision of going to hospital a lot is based on fear, because of the [potential] repercussions … The main reason [patients nearing end-of-life] end up going to A&E [accident and emergency department] is because of lack of information … You would end up taking that patient to A&E, even though it feels wrong … information is a form of support. It supports your decision-making whilst you are on the scene. [Lack of information is] always a barrier to making the best decisions for the patient. (Paramedic6).

These accounts emphasise that limited access to information is a significant factor influencing paramedics’ decision to facilitate community-based care. In the next section we summarise the views of paramedics on a potential solution to this problem: an EPaCCS.

### Views on the proposed EPaCCS

Overall participants expressed enthusiasm about the possibility of accessing an electronic record encompassing patient information documented by various health and social care professionals. Key information that interviewees felt would be useful to access through the proposed EPaCCS included acknowledgement that the patient was on the end-of-life care pathway and links to their ACP documentation, or details of where to find the patient-held paper copies. Other factors that were frequently mentioned included: medical history, clinical observations, and contact details for individuals providing community-based care for the patient. The ability to remotely access such information, prior to and while tending to the patient, was recognised to have several potential benefits for paramedics, including: improving their decision-making (in terms of speed and quality), supporting them to arrange community-based care more frequently, and improving their confidence in managing the care of patients nearing end-of-life:

I do think there are a number of paramedics out there who might, with [a shared electronic record] in front of them, make better informed decisions for that episode of patient care. Which would ultimately be better for the patient, but could also, as another aspect, help them grow and develop in confidence in dealing with these kinds of patients as well. (Paramedic8).

While there was little doubt that access to an EPaCCS could improve the ability of paramedics to provide care in line with patient’s wishes, interviewees recognised several practical and technical issues that could pose challenges. The most prevalent issue concerned the reliability of mobile devices and electronic systems. Existing technology and IT systems used by paramedics were felt to be fallible, working intermittently or not at all, particularly in rural areas with poor access to Wi-Fi or telephone signal. Interviewees expressed concerns that this could limit access to the electronic record creating new, electronically determined, information blind spots:

… if we’ve got no wireless or mobile data [the mobile device] doesn’t work, so how will I pick up the patient records? [… Technology] doesn’t work out in the sticks. Therefore what are we doing? We’re not providing an inclusive care, are we? The rural people are going to give it, “Well this isn’t fair.” Perhaps arguably it isn’t. (Paramedic3).

Other potential issues raised by paramedics included: record accuracy, time taken to access and understand the information, and professional resistance to engage with the system:

[If] you go in but there’s plainly an issue that needs sorting now, who’s going to look at the patient data? [… An electronic record] would be beneficial, but you’ve got to have the time to access it, and technical competency and ability to actually drag the data out […] you’re going to get a reasonable amount of people that view it as an extra inconvenience, I suppose, an extra hassle …. If people still wanted to take the Luddite approach, you could not access it and do exactly the same as you’re doing now. But that’s not better for anyone, really, is it? (Paramedic2).

Further to this, some interviewees acknowledged that even if these issues could be resolved, access to an EPaCCS would not always facilitate the provision of community-based care, as paramedics sometimes lack access to the equipment or technical knowledge to deliver this:

If I go to someone and they need pain relief, unless I’ve got the syringe drivers or the drugs to leave at home, I’ve got to go with plan B and take them to the hospital. So you’re only as good as your training and the equipment and pathways that you have available to you. And you’ve got to have all of those elements. (Paramedic4).

This view emphasises that while streamlining access to detailed patient information through an EPaCCS could improve the way paramedics manage the care of patients nearing end-of-life, this is one of a series of changes that must occur in tandem if staff are to facilitate community-based care at the end-of-life.

## Discussion

This study explored the extent to which access to, and quality of, patient information affects the care that paramedics provide to patients nearing end-of-life. Reflecting previous work, our findings reveal that the limited information available to paramedics means they operate at a disadvantage compared to other healthcare professionals involved in end-of-life care [2, 5]. Limited access to accurate and in-date ACP documentation was reported to have a significant impact on the care provided by paramedics [3, 5, 28]. The perceived worth of, and paramedic willingness to comply with, ACP documentation was challenged by the fact that these forms are routinely out-of-date and/or felt to be too ambiguous to be useful in an emergency [6]. As has been reported in research from other countries (including New Zealand and the US) [14, 15], our study identified that poor access to valid and robust information on patients nearing end-of-life generated a sense of uncertainty among paramedics about the best course of action, and subsequent fear about potential litigation. This had a significant influence on how confident paramedics felt in their decision-making, leaving them hesitant about facilitating community-based care and more likely to transfer the patient to hospital, a decision often felt to be inappropriate and/or contradictory to the patient’s wishes or interests [1, 3, 5].

Recently published research calls for studies to consider and assess potential ways to address the issues faced by paramedics providing end-of-life care, including limited access to patient information [5]. Paramedics in this study regarded the proposed EPaCCS as a positive initiative with the potential to improve patient care at end-of-life, particularly with regards to the speed and quality of decision-making. They postulated that this would aid professionals access to appropriate data, working towards the aims of the aforementioned NHS Framework for Action [21]. Though improving access to patient information through an EPaCCS was generally felt to be a step in the right direction, paramedics recognised that this must occur as part of a wider series of practice changes, if the provision of community-based care for patients nearing end-of-life is to become routine practice. Other changes viewed as key to bolstering paramedics’ autonomy when providing end-of-life care included improved access to resources (e.g. equipment and anticipatory medication) and specialised training. While improvements to the information, training, and resources available to paramedics would equip them to provide better end-of-life care, implementation of such change on a national scale is likely to prove very challenging, as many of the underlying issues - financial, cultural, and organisational – are longstanding and extend beyond the ambulance service [5, 29]. Significant transformation of the way the NHS operates is required, particularly with regard to the organisational culture, to ensure initiatives aimed at improving end-of-life care (including those discussed within this paper) are worthwhile and meaningful [30–32]. The NHS are aware of and working to address such issues, however, the scale and complexity of the organisation means it is inevitable that this process will take a significant amount of time [31, 32].

The findings of this study provide useful insight for professionals within and beyond the UK, as healthcare systems around the world increasingly recognise the importance of ACP in end-of-life care [33–35] and the need to implement electronic patient records to facilitate the delivery of appropriate care [19, 20]. This study has identified a need, and strong desire, for improved access to accurate and in-date ACP documentation for paramedics attending patients at end-of-life. Access to an EPaCCS offers a potential solution, however, several barriers need to be addressed for this to be successful in practice. Further research is needed to support the development and implementation of EPaCCS for paramedics, and to evaluate its usefulness in practice. As the success of such a record depends on the quality and consistency of the information uploaded, additional research exploring, and training provided to improve, professional recording practices in end-of-life care is vital to aid the establishment of an effective record.

### Limitations

The findings reported within this paper are rooted in data collected from a small number of interviewees (predominantly male, all white-British) from one UK-based ambulance service and thus must be interpreted with caution. It is possible to argue that the wider generalisability of these findings is limited by the relatively small and locality-specific nature of the sample, however, it is important to bear in mind the considerable size and geographically diverse nature of the region served by this ambulance service. As many of the findings discussed in this paper reflect those documented elsewhere in the UK, it is reasonable to suggest that they are likely  to have relevance beyond this sample. As this study provides novel insight on paramedic views of a shared electronic record, it is likely to prove highly relevant as practice moves toward the increased use of electronic records, in the UK and elsewhere [19, 20].

## Conclusion

Greater access to accurate and in-date patient information, particularly ACP documentation, is required to support paramedics in their decision-making processes, and enable them to facilitate community-based care when appropriate and sought by the patient. Access to an EPaCCS offers a solution to the inconsistent availability of information, however, several barriers exist which must be addressed to ensure the success of such a record in practice. While access to ACP documentation is evidently a key issue preventing their implementation among paramedics, there are other influential factors at play, such as paramedic unwillingness or inability to comply with the plan. Improving access to patient information is not the only change required to enhance paramedic provision of end-of-life care, issues with equipment availability and technical expertise must also be addressed.

## Data Availability

The datasets generated and/or analysed during the current study are not publicly available to preserve the anonymity of participants. Releasing data would be an infringement of our ethics.

## Abbreviations

ACP: Advance Care Planning
DNACPR: Do not attempt cardiopulmonary resuscitation
EHCP: Emergency Health Care Plan
EPaCCS: Electronic Palliative Care Coordination System
